# Evaluation of Three Hydration Strategies in Detection Dogs Working in a Hot Environment

**DOI:** 10.3389/fvets.2017.00174

**Published:** 2017-10-26

**Authors:** Cynthia M. Otto, Elizabeth Hare, Jess L. Nord, Shannon M. Palermo, Kathleen M. Kelsey, Tracy A. Darling, Kasey Schmidt, Destiny Coleman

**Affiliations:** ^1^Department of Clinical Studies, School of Veterinary Medicine, University of Pennsylvania, Philadelphia, PA, United States; ^2^Penn Vet Working Dog Center, School of Veterinary Medicine, University of Pennsylvania, Philadelphia, PA, United States; ^3^Dog Genetics, LLC, Sunnyside, NY, United States; ^4^College of Veterinary Medicine, Washington State University, Pullman, WA, United States

**Keywords:** canine, hydration, search, behavior, sodium, electrolytes, heat stress

## Abstract

Physical activity in hot environments can increase the risk of heat stress or heat stroke in dogs. Heat tolerance is influenced by acclimatization to the environment, physical fitness, and hydration state. Three common strategies to promote hydration in working dogs are free access to water (W), oral electrolyte solutions (OESs), and administration of subcutaneous fluids (SQs). None of these methods have been compared for safety or efficacy in a working environment. In a cross-over design, seven vehicle-screening canines were randomly assigned to each of the three hydration strategies during working shifts at the Sarita, TX checkpoint. Physical, behavioral, and biochemical parameters were collected before, during, and after a work shift (mean 5.7 ± 0.8 h). Dogs were given 10 mL/kg oral W, 10 mL/kg chicken flavored OES, or 15 mL/kg of SQs initially followed by controlled access to W or OES. The dogs drank 15.61 ± 4.47 mL/kg/h of W and OES when in the OES group, compared to 7.04 ± 3.42 and 5.56 ± 4.40 mL of W, for the W and SQ groups, respectively. The median environmental temperature was 84.8°F (29.3°C). The median humidity was 70%. Based on mixed effects linear modeling, dogs in the OES and SQ groups had significantly higher total CO_2_, and lower packed cell volume and total plasma protein at the end of the day. Creatinine increased a small but significant amount in the SQ group and decreased in the OES group. Searching behaviors were independent of hydration strategy but highly related to the dog specific factors of sex, breed, and activity level. Under conditions of controlled activity in moderate heat and humidity, dogs accustomed to the work and the environment were more likely to increase fluid consumption and hydration when provided a flavored OES. Potential benefits of OES and SQ were indirect and no adverse effects were documented for any of the hydration strategies tested.

## Introduction

Military and other working dogs (e.g., police, search-and-rescue) are critical to protect our national security and respond to disasters. Working dogs are expected to perform physically and mentally demanding tasks often in adverse environmental conditions. Many working dogs are highly motivated to perform the tasks of searching or criminal apprehension and may exert themselves to the point of severe dehydration, collapse, heat stress, and even heat stroke (1–4). One of the most common potentially preventable causes of death in military working dogs is heat stroke (2). The risk of heat stroke increases with dehydration (5). Dehydration can be a complication of these intense working environments. Even in the moderate temperatures that occurred in September of 2001, handlers reported dehydration in the search dogs working at the World Trade Center and Pentagon (6). Dehydration was the most common medical finding in dogs deployed to the 2010 earthquake in Haiti (7). Austere environments, such as war zones in the Middle East and border regions in the southern US, further increase the risk of dehydration.

Since dogs have minimal sweating capacity, thermoregulation relies primarily on evaporative mechanisms through panting (8). Heat, humidity, and hydration are thought to impact the dog’s ability to thermoregulate (4, 9, 10). High ambient temperatures decrease the heat gradient from the dog to the environment and add external heat to the dog. Humid conditions impair effective evaporative cooling. Humid environments, such as those experienced in response to Hurricane Katrina and in US Border regions along the Gulf Coast, can contribute to heat stress. In addition to the environmental factors of heat and humidity, the dog’s adaptation to environmental conditions (also called acclimatization), overall fitness/conditioning and state of hydration are thought to be major factors that impact heat tolerance (11). Canine thermoregulation involves the inhalation of cooler, dryer air through the nose and mouth, which causes evaporative heat loss from the nasal and oral mucosa and tongue, and the exhalation of hotter, moister air. In conditions in which the rate of heat generation is greater than the rate of heat dissipation, increased salivation and lingual blood flow allow for convection and evaporative cooling (12). Evaporative cooling results in electrolyte-free water loss (estimated at ~10 mL/kg/h) which can contribute to dehydration (13). Most studies of working or hunting dogs have shown small or no electrolyte changes following exercise (4, 14, 15). In one study of sled dogs, sodium was significantly decreased following 10 days of endurance racing (16, 17). Because dogs do not sweat, any electrolyte loss would be through saliva or urine. Salivary Na and Cl are lower than the serum, and salivary bicarbonate, K, and Ca are higher (18). In hydrated dogs, salivary loss is estimated at 7 mL/kg/h during exercise (13). In dehydrated dogs, the amount of water lost to cooling was approximately 7% lower than in hydrated dogs, but salivation was reduced by over 90% (13). Increased urinary sodium loss was hypothesized in hyponatremic sled dogs despite an inability to document it at the sampling time after the race (15). As dehydration increases, the availability of water for evaporation, salivation, and circulation will be diminished; heat tolerance and physical, mental, and olfactory performance will be reduced.

Conditioned human athletes are better able to tolerate heat than non-athletic humans (19). Oxygen utilization (VO_2_max) is a classic measure of cardiovascular fitness. In dogs, VO_2_max measurement has been limited to research studies (20, 21). Lactate threshold has been used as a surrogate for VO_2_max, but is not widely utilized in canine exercise physiology (21). A 6-min walk test has been used to differentiate obese dog cardiopulmonary performance from lean dogs, but did not discriminate between overweight and lean dogs (22). In an exercise conditioning study of police dogs, conditioned dogs had higher post exercise temperatures and lower creatine kinase and aspartate aminotransferase (AST) (23). There is no standard test to define fitness in canine athletes in the field or evaluate the impact of physical conditioning on heat tolerance in dogs. Both acclimatization and conditioning are long-term management strategies that should be considered, though in the acute setting, manipulating hydration is the most appealing approach to prevent dehydration and increase heat tolerance and workability.

Hydration strategies employed may vary with the location and the nature of the mission. Currently, dog handlers commonly use oral water (W), subcutaneous fluids (SQs), and oral electrolyte solutions (OESs) to prevent dehydration. Neither the safety nor efficacy of these hydration strategies has been previously evaluated. The aim of this study was to evaluate the effects of three hydration strategies on hydration status and performance in Border Patrol dogs screening vehicles on the Texas border in the summer. We hypothesized that there would be no difference between the OES, W, and SQ groups for hydration parameters or adverse effects.

## Materials and Methods

### Animals

Seven Border Patrol dogs working for Customs and Border Protection at the Sarita, Texas border station (Rio Grande Valley Sector) were selected based on canine and handler availability during the experimental period. All dogs were trained to screen vehicles and to alert to both narcotics and concealed live humans. All dogs worked, trained, and were kenneled at the same facility, and all lived with their handlers. All dogs were deemed healthy and in good condition based on physical examinations. The age, breed, sex, neuter status, physical examination parameters, and history were collected from the handlers on all dogs.

### Experimental Design

All protocols and the study were approved by the University of Pennsylvania and US Army Medical Research and Materiel Command Institutional Animal Care and Use Committees (U Penn IACUC protocol 804293, USAMRMC proposal SO120002) and all experimental procedures were conducted in accordance with the recommendations of the committees. In this field study where dogs were working their normal assigned shifts, each dog was randomized independent of the other dogs to account for differences in work schedules. Dogs were randomly assigned to one of three treatment protocols on each of 3 days of study participation. Study days were limited to Monday, Tuesday, Wednesday, and Thursday to eliminate the uncontrolled variable of higher traffic on weekends. Only dogs scheduled for duty between 7:00 a.m. and 2:00 p.m. or between 9:00 a.m. and 3:00 p.m. were included. During this time period, dogs typically worked 30 min then rested 30 min. Dogs were studied in groups of 2 or 3 dogs on the same day, depending on handler schedule. Based on a pilot study performed with the Philadelphia Police Canine Unit, dogs initially received 10 mL/kg oral W, 10 mL/kg OES (Hydrolyte, Advanced Nutritional Support, Elka Park, NY, USA), or 15 mL/kg of SQs (Plasmalyte A, Abbott Laboratories, North Chicago, IL, USA) initially. See Table 1 for the electrolyte composition of the supplements. Every 30 min, whether they were working or resting, dogs in each treatment group were offered 10 mL/kg of W. If the dogs in the OES group drank less than 3 mL/kg of water, they were offered 10 mL/kg of OES. W and OES were kept in an air-conditioned room prior to offering it to the dogs. The dogs in the W and SQ groups were only offered water. If the dogs consumed the entire 10 mL/kg of water before the end of the 30-min time interval, they were offered a second 10 mL/kg bowl.

**Table 1:** Measured and reported electrolyte composition of oral electrolyte solution (OES) and subcutaneous fluids (SQ).

Ingredient	OES measured	SQs reported
Sodium (mmol/L)	152	140
Potassium (mmol/L)	7.1	5
Chloride (mmol/L)	109	98
Buffer	Bicarbonate 25 mmol/L	Acetate 27 mEq/L
		Gluconate 23 mEq/L
Magnesium (mmol/L)	2.3	1.5
Glucose (mmol/L)	13.7	0
Osmolality (mOsm/L)	332^a^	294
Effective strong ion difference (mEq/L)^b^	50	47

*^a^Osmolality was calculated as 2([Na^+^] + [K^+^]) + ([glucose]/18), where brackets represent concentration*.

*^b^Effective strong ion difference = [Na^+^] + [K^+^] − [Cl^−^]*.

The time of each hydration interval and study activity was recorded for each dog. If any dog exhibited signs of physical distress (i.e., anxiety, aggression, lethargy, unwillingness to work) and/or was unable to maintain adequate hydration, it was to be removed from the study and treated appropriately.

### Data Collection

#### Timing of Data Collection

A physical examination was performed on each dog at the beginning of each day. Body weight, temperature, pulse, respiratory rates, urine, and blood samples were obtained at the beginning, middle, and end of each work shift.

Fluid volume consumed, internal body temperature, heart rate and rhythm, qualitative assessments of activity, urination, and defecation, as well as ambient temperature, percent humidity, and wind speed, were measured every 30 min.

#### Dog Fluid and Food Intake and Urination/Defecation

The fluid consumption was measured based on the remaining fluid left in the bowl. The bowls were held as each dog drank to minimize spillage, however there was no way to account for small amounts of fluid that was splashed by the dog. The total fluid volume, fluids administered for the SQ group, water intake for the SQ and W groups, and water plus OES for the OES group, was recorded for each dog for each study day and normalized to the body weight and duration of work (mL/kg/h). Sodium load was calculated based on the sodium content of each fluid (Table 1) and the volume of each fluid. A subjective measure of urine volume (increased, decreased, or normal) and fecal characterization based on the handler’s knowledge of his dog’s normal elimination patterns and fecal scoring using the 1–7 score with accompanying photographs of examples (24, 25) was recorded for each dog every 30 min during each study day. A veterinarian was on site to confirm any reported abnormal fecal scores during the work shift. Dogs were maintained on their normal feeding schedule; they were not fed during the work day, except for a small amount of canned dog food associated with the ingestion of the internal temperature sensing capsules of the CorTemp^®^ system (HQInc Wireless Sensing Systems & Design; Palmetto, FL, USA).

#### Dog Activity

Quantitative activity counts were monitored using omni-directional accelerometers (ActiCal, Philips Respironics; Murrysville, PA, USA) (26). During the work day, each dog wore an activity monitor programmed with its identity. The activity monitors were secured to standard flat buckle collars upon arrival onsite each morning and removed after the end of each day’s final search. At the end of each study day, data were transferred from the monitors to a computer using the ActiReader data-downloading device. Total activity counts were normalized for hours of work.

Qualitative activity levels and location of activity were recorded every 30 min based on an expected cycle of search and rest. If any other activity, such as a secondary search, was performed, or a subject performed more than one activity in the 30-min interval, the activities were noted, accompanied by the approximate time spent performing each activity.

#### Internal Body Temperature

Internal (gastrointestinal) body temperature was monitored using the CorTemp^®^ system (27). On the first day of participation in the study and after morning weight was obtained, each dog was administered a non-toxic ingestible core body thermometer pill placed in approximately 5 tablespoons of canned dog food (Pedigree, Mars Global Pet Care, Franklin, TN, USA). In the mornings of subsequent study days, dogs were scanned for the CorTemp^®^ capsule prior to being offered another capsule. If the original capsule was present in the dog and transmitting appropriately, a new capsule was not administered until the old capsule passed. Body temperatures were recorded every 30 min with a handheld CorTemp^®^ Data Recorder. If a reading could not be obtained due to capsule or recorder error, rectal temperatures were taken instead. At the end of the study, CorTemp^®^ data were downloaded from the Data Recorder onto a computer using the CorTrack™ II software. For analysis, the difference between the values collected at baseline and at the end of the day was utilized.

#### Heart Rate and EKG

Heart rates and EKGs were monitored using the AliveCor iPhone 4 s phone case and iPhone app (AliveCor, Inc., San Francisco, CA, USA) as validated for cats and dogs (28). Heart rates were recorded every 30 min with the AliveCor device. The electrodes on the phone made contact via isopropyl alcohol with the thorax between rib spaces 3–7 for approximately 10–60 s to obtain an accurate heart rate and EKG recording. Any cardiac abnormalities were noted. If an EKG reading was unable to be obtained, pulse or heart rates were obtained by femoral pulse palpation and/or cardiac auscultation. For analysis, the difference between the values collected at baseline and at the end of the day was utilized.

#### Body Weight, Blood Samples, and Urine Measurements

Weight in kilograms was obtained using a walk-on electronic scale (Jorvet J0825PM, JorVet Walk on Scale 36″; Jorgensen Labs, Loveland, CO, USA) that was calibrated twice daily. Blood samples (3 mL) were obtained from a peripheral or jugular vein and were directly analyzed or anticoagulated in a Li heparin vacutainer for use in an ISTAT CHEM8^+^ to measure sodium (Na^+^), potassium (K^+^), chloride (Cl^−^), ionized calcium (iCa), total CO_2_ (TCO_2_), glucose (Glu), blood urea nitrogen (BUN), creatinine (Creat), hematocrit (Hct), hemoglobin (Hb), and anion gap (Abaxis veterinary research laboratories, Union City, CA, USA). Lactate blood levels were analyzed directly using a handheld lactate meter (Lactate Scout, EKF Diagnostics, Penarth, Cardiff, UK). All remaining blood was placed in an EDTA tube and four samples were removed and placed in microhemotocrit tubes and centrifuged at 11,000 rpm for 3 min (LW Scientific ZIPocrit Centrifuge, Lawrenceville, GA, USA) to obtain packed cell volume (PCV). A handheld refractometer, was used to determine total protein (TP). All remaining blood in the EDTA tube was centrifuged at 3,150 rpm for 5 min and the plasma was collected and frozen for future analysis. Urine specific gravity (USG) was measured onsite with a handheld refractometer and urine microalbumin was measured with a commercially available semiquantitative test strips (Heska E.R.D. Healthscreen Canine Urine Tests, Heska, Loveland, CO, USA) on free catch midstream urine samples. Urine samples were stored at 4°C for sodium and Creat analysis by the Vitros 4600 Chemistry System (Ortho Clinical Diagnostics, Raritan NJ). Briefly, Creat is hydrolyzed to creatine in the rate-determining step. The creatine is converted to sarcosine and urea by creatine amidinohydrolase. The sacrosine, in the presence of sarcosine oxidase, is oxidized to glycine, formaldehyde, and hydrogen peroxide. The final reaction involves the peroxidase-catalyzed oxidation of leuco dye to produce a colored product. The resulting change in reflection density is measured at two time points and the difference in reflection density is proportional to the concentration of Creat present in the sample. For Na, the slide consists of two ion-selective electrodes, each containing methyl monensin (an ionophore for sodium), a reference layer and a silver layer and a silver chloride layer coated on polyester support. A drop of patient sample and a drop of VITROS reference fluid on separate halves of the slide results in migration of both fluids toward the center of the paper bridge. The liquid enters the slides and a stable liquid junction is formed that connects the reference electrode to the sample electrode. Each electrode produces an electrochemical potential in response to the activity of Na. The potential difference between the two electrodes is proportional to the Na concentration in the sample. For analysis, the difference between the values collected at baseline and at the end of the day was utilized.

Fractional excretion of sodium was calculated as described by Hinchcliff (16)

$${\text{FC}}_{\text{Na}}=\left({\text{U}}_{\text{Na}}*{\text{S}}_{\text{creat}}\right)/\left({\text{S}}_{\text{Na}}{\text{U}}_{\text{creat}}\right)*100,$$

where U_Na_ and S_Na_ are the concentrations of sodium in urine and serum, respectively, and S_creat_ and U_creat_ are the concentrations of Creat in serum and urine, respectively.

#### Odor Detection Performance

**Figure 1:**
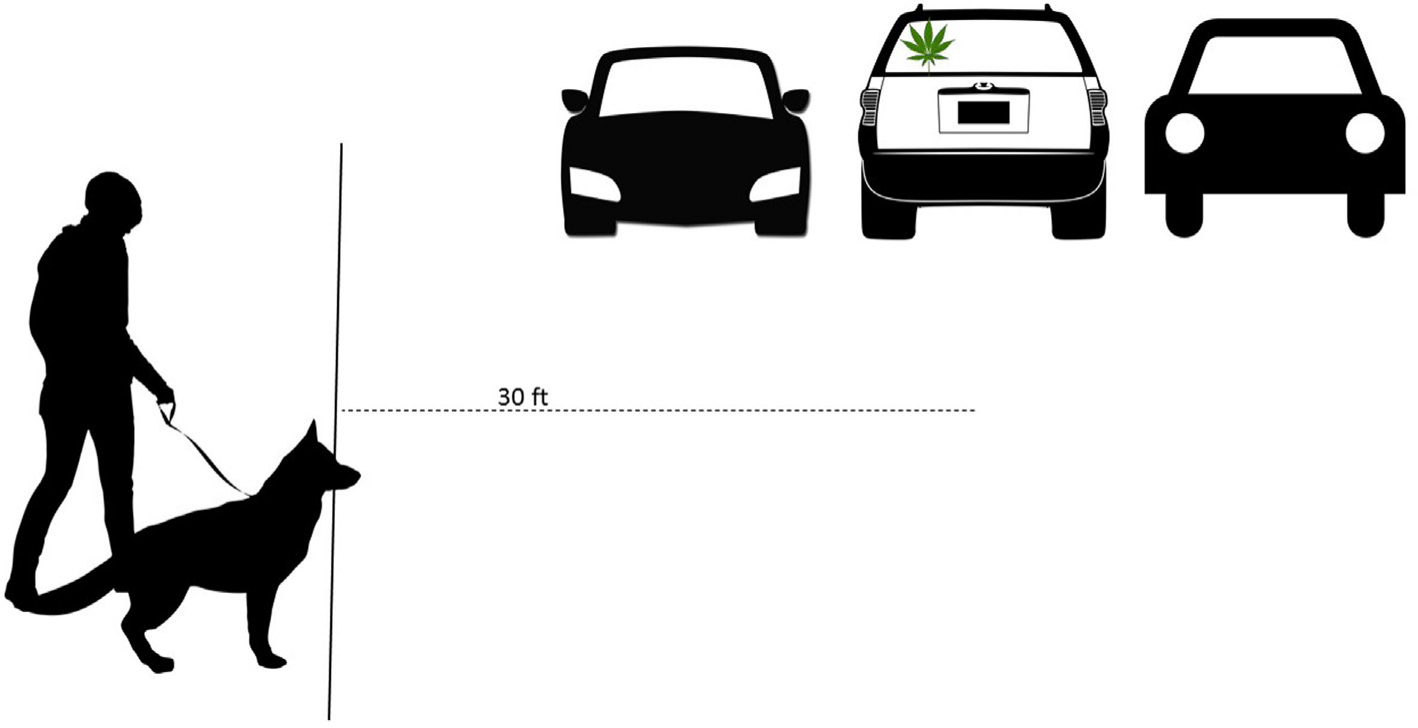
A diagram representing the standardized search activity. The handler and the dog were positioned 30 feet from the vehicle containing the concealed training aid (signified by the marijuana leaf). The dog searched in a detailed fashion on leash until it alerted to the training aid.

A standardized search problem was set up at the beginning and end of each workday (see Figure 1; Video S1 in Supplementary Material depicting a training session in which a dog and a handler team conduct a detailed vehicle search). A training aid containing a scent that the dog was trained to find or a live human was hidden in a vehicle. The training aid/human was in the same location for all dogs at a single time point on a given day, but the location was different on each day and different in the morning and the evening to minimize handler influence. The search was set up to mimic the format of a secondary road search of a parked vehicle at the checkpoint. The distance from the starting point to the middle of the car in which the training aid was hidden was 30 feet, and this information was not provided to the handlers. Starting points were adjusted based on the location of the training aid. The number of cars and/or other obstacles varied from day to day but contained no less than 1 vehicle and no more than 3 vehicles. The handler was not informed of the location of the training aid. The time from initiation of search at the starting point to the time of indication was recorded. Any false alerts or behavior changes were recorded. The location of the training aid was selected by the canine instructor, and the training aid was placed at least 5 min prior to the search start. Each search was recorded using both a fixed and a handheld video camera (Sony Handicam Camcorder HDR-CX380/B, Sony Corp., New York, NY, USA). Ambient temperature, humidity, wind speed, and wind direction were recorded with both a handheld and wireless device (Extech Anemometer/Thermometer/%Humidity, Extech Instruments, Nashua, NH, USA; and AcuRite Wireless Weather Station; Primex Family of Companies, Lake Geneva, WI, USA). The video recordings were reviewed using Noldus Observer Software (Noldus Information Technology, Leesburg, VA, USA) by a single reviewer that had not participated in the field study. The duration of behaviors was recorded. The behavioral ethogram (see Table 2) was derived from different ethograms evaluating stress in dogs under various environments (29–31). Each behavior was reported as the proportion of the search time in which it was observed. For analysis, the frequency of behaviors in the morning search for each dog was subtracted from the frequency of that dog’s behaviors in the afternoon search.

**Table 2:** Behaviors recorded.

Ear position	Up
	Down
Ear state	Relaxed
	Alert
Tail position	Tail up
	Tail down
Tail movement	Wagging
	Stiff
	Relaxed movement
Mouth state	Relaxed
	Taut
Mouth position	Open
	Closed
Mouth activity	Licking
	Panting
Tongue position	In mouth
	Out to side
	Out straight
Canine focus	On handler
	On search
Post-search reward	Tug play
	Other reward
Interactions with canine	Cueing the location of the aid
	No reward for alert
	Successful alert and reward
	Dog jumping up on handler during reward
	Placing feet up on handler during reward
	Loose grip on the reward tug toy
	Strong grip on the reward tug toy

### Statistical Analysis

Descriptive data were visually inspected and tested for normality using Kolmogorov–Smirnov test in a commercial statistics package (SigmaPlot 11.0, Systat Software; Chicago, IL, USA). Data were reported as mean and SD for normally distributed data or median and range for nonparametric data.

Mixed-effects linear models were fit using the lmer function in the lme4 (32) package in the open-source R statistical software package which is available, along with supplementary packages, at https://cran.r-project.org. This function fits mixed models to data with crossed factors and is robust to deviations from the normal distribution. It estimates variance parameters for a data set using an iterative maximum likelihood procedure that searches for a set of variance estimates that have the highest probability (likelihood) of producing the data (33).

The serum chemistry dependent variables tested were lactate, sodium, potassium, chloride, iCa, total bicarbonate, Glu, Creat, and anion gap. The hematology-dependent variables tested were hematocrit, PCV, TP, and Hb. Other dependent variables tested were the change in weight, change in pulse, and fractional excretion of sodium over the day of work. For the full model, fixed factors included hydration strategy, sex, and breed and individual ID was a random factor. Covariates included activity count (counts/h), dog’s change in weight (kg), age in months, change in pulse (maximum–minimum), liquid intake (mL/kg/h), change in USG (morning–afternoon), average daily ambient temperature (F), and average daily humidity. Age was fitted as a covariate to account for variation between dogs and because covariates provide more statistical power than age categories fitted as fixed effects. For the dependent variable fractional excretion of sodium, an additional covariate, sodium load, was included in the model, and intake of liquid including SQ fluids was used (mg/kg/h). To address warnings from the maximum likelihood procedure about variables being of different scales, activity counts/h was divided by 10,000, age in months was divided by 10, and USG was multiplied by 100.

The drop1 function was used to assess the significance of each fixed factor and covariate in contributing to the fit of the model. drop1 fits a set of models with each model missing one of the variables present in the original model. The significance of a variable is determined by a chi-square test of the likelihoods of the new model (missing the relevant variable) relative to the original model. Fixed factors and covariates with *p* < 0.05 for the chi-square test were included in a refined model. This process was continued until all the variables were significant according to drop1, with a final model fitted including the significant drop1 variables from the previous model. The reported *p*-values for variables were obtained using the ANOVA function in the “car” package. The pairs function of the lsmeans package in R was used to obtain contrasts of hydration methods and confidence intervals for hydration strategies when they contributed significantly to the fit of the model. The baseline value for the contrasts of hydration strategies was W so that both OES and SQ strategies could be compared with the method most commonly in used. R’s summary function was used for each refined model to determine the regression coefficient and significance of factors such as hydration strategy.

The same model selection method was used to model fractional excretion of sodium except that total fluid, including liquid administered subcutaneously replaced total liquid intake, and sodium load was included as an additional covariate.

Mixed effects models were also used to determine whether hydration strategy was associated with scores on behavior measures such as ear, tail, mouth position, attention, and interactions with the handler. Models for behavior scores included hydration method, sex, and breed as fixed factors, change in body weight, activity level, ambient temperature, and humidity as covariates, and individual as a random factor. The same procedure of dropping variables from the models and retaining significant ones was used to arrive at final models.

Based on the mixed model, fractional excretion of sodium was not influenced by dog or by treatment strategy; therefore, data were combined for all dogs and all groups. The effect of time of day was evaluated using Mann–Whitney rank sum test.

## Results

Three females and four males participated. Two females were spayed Belgian Malinois, and one female was a spayed German Shepherd Dog. Of the males, two were neutered Belgian Malinois (one short coated, one long coated), and the intact males included a Belgian Malinois and a German Shepherd Dog. All dogs were between the ages of 3 and 8 years of age, with a mean age of 5.5 ± 1.9 years. Mean weight was 30.6 ± 4.3 kg.

The mean internal body temperature across all test days was 101.9 ± 1.3°F (38.8 ± 0.7°C). The maximum temperature recorded was 104.5°F (40.3°C) and only five recordings (representing three dogs) were ever greater than 103.0°F (39.4°C). The median heart rate was 100 beats/min with a range of 96–141. No arrhythmias were detected. During the majority of examinations, dogs were panting.

Daily examinations did not find any medical problems that precluded work for any of the dogs. All dogs completed each study day. One dog ended one study day early due to the handler’s personal emergency. No dog had abnormal stool consistency. Of the 97 reports on urine production, the majority (54) described normal volume, 32% were decreased volume and 11% were increased volume. Each of the three hydration strategies was represented in each of the subjective assessments of urine volume. Mean USG prior to commencing with work was 1.031 ± 0.014; whereas mean USG at midday was 1.022 ± 0.013 and at the end of the day median USG was 1.027 (range 1.003–1.042). No microalbumin was detected in any urine sample at any time. Other than the food associated with delivery of the internal temperature sensing capsule, no dog was fed during the work shift. The mean and SD of the chemistry, hematologic, urine, and physical variables as a function of time and group can be found in Tables 3–6.

**Table 3:** (A) Plasma chemistry results as a function of time and treatment group in the water (W) group, (B) plasma chemistry results as a function of time and treatment group in the oral electrolyte solution (OES) group, and (C) plasma chemistry results as a function of time and treatment group in the subcutaneous fluids (SQs) group.

	AM		Midday		PM	
	Mean	SD	Mean	SD	Mean	SD
**A**	**W**					

Lactate (mmol/L)	0.119	0.033	0.095	0.038	0.088	0.018
Na^+^ (mmol/L)	146.14	1.95	146.00	0.82	146.57	2.15
K^−^ (mmol/L)	4.04	0.45	3.96	0.3	3.91	0.46
Cl^−^ (mmol/L)	117.14	2.34	117.57	2.07	117.57	2.64
iCa (mmol/L)	0.348	0.023	0.343	0.023	0.338	0.023
TCO_2_ (mmol/L)^a^	19.6	2.6	18.7	1.7	17.4	1.3
Glucose (Glu; mmol/L)	5.10	0.52	5.04	0.56	5.06	0.30
BUN (mmol/L)	6.32	3.78	5.71	3.55	5.20	3.01
Creat (μmol/L)^b^	79.56	15.91	84.86	18.56	87.52	11.49
Anion gap	14.6	1.5	14.6	1.9	16.4	3.6

**B**	**OES**					

Lactate (mmol/L)	0.127	0.037	0.133	0.072	0.108	0.030
Na^+^ (mmol/L)	145.43	4.04	146.71	1.11	145.83	4.26
K^−^ (mmol/L)	4	0.4	3.71	0.43	4.72	2.13
Cl^−^ (mmol/L)	118.29	2.87	117.14	2.48	116.83	2.86
iCa (mmol/L)	0.348	0.015	0.333	0.030	0.335	0.013
TCO_2_ (mmol/L)^a^	19.6	1.4	19.3	2.8	20.7	1.6
Glu (mmol/L)	5.35	0.49	5.14	0.34	4.87	0.65
BUN (mmol/L)	6.38	3.22	5.15	2.02	5.12	2.66
Creat (μmol/L)^b^	80.44	16.80	80.44	10.61	83.98	12.38
Anion gap	14.7	1.4	14.7	2.3	15.2	0.8

**C**	**SQs**					

Lactate (mmol/L)	0.110	0.046	0.100	0.041	0.110	0.067
Na^+^ (mmol/L)	146.29	2.29	145.86	3.53	145.43	1.13
K^−^ (mmol/L)	4.07	0.4	3.7	0.41	3.84	0.48
Cl^−^ (mmol/L)	117.71	1.7	117.71	1.89	118.14	2.27
iCa (mmol/L)	0.340	0.025	0.328	0.035	0.338	0.035
TCO_2_ (mmol/L)^a^	20.0	1.8	19.1	0.9	18.3	2.2
Glu (mmol/L)	5.10	0.54	5.13	0.29	5.02	0.44
BUN (mmol/L)	5.97	2.56	5.05	1.78	4.64	1.64
Creat (μmol/L)^b^	80.44	14.14	84.86	9.72	88.40	12.38
Anion gap	0.110	0.046	0.100	0.041	0.110	0.067

*All values are reported as the mean and SD in international units*.

*iCa^+^, ionized calcium; tCO_2_, total carbon dioxide; BUN, blood urea nitrogen; Creat, creatinine*.

*^a^For TCO_2_, the change over the day was significantly different for W versus OES (*p* < 0.0001) and W versus SQ (*p* < 0.0001)*.

*^b^For Creat, contrasts were significant between all three pairs of hydration types: W and OES (*p* = 0.015), W and SQ (*p* = 0.010), and OES and SQ (0.001)*.

**Table 4:** (A) Hematology results as a function of time and treatment group in the water (W) group (B), hematology results as a function of time and treatment group in the oral electrolyte solution (OES) group, and (C) hematology results as a function of time and treatment group in the subcutaneous fluids (SQ) group.

	AM		Midday		PM	
	Mean	SD	Mean	SD	Mean	SD
**A**	**W**					

Hct (%)	45.3	4.5	42.9	2.9	41.3	4.8
PCV (%)^a^	49.3	4.8	47.4	3.1	45.0	3.7
TP (g/L)^b^	71.3	3.1	69.4	2.9	68.9	3.2
Hgb (g/L)	152.4	16.1	145.9	9.8	140.3	16.2

**B**	**OES**					

Hct (%)	45.4	3.5	41.0	2.7	38.3	1.8
PCV (%)^a^	48.7	3.0	46.1	3.0	43.5	2.2
TP (g/L)^b^	71.0	2.5	66.9	2.5	66.5	4.4
Hgb (g/L)	150.6	12.8	139.3	9.1	130.3	6

**C**	**SQs**					

Hct (%)	44.3	3.9	43.1	3.3	41.3	3.7
PCV (%)^a^	48.6	3.8	47.1	3.5	45.3	2.9
TP (g/L)^b^	70.6	3.7	70.4	3.6	70.0	3.4
Hgb (g/L)	150.6	13.2	146.7	11.2	140.4	12.4

*All values are reported as the mean and SD in international units*.

*Hct, hematocrit; PCV, packed cell volume; TP, total protein; Hgb, hemoglobin*.

*^a^For PCV, contrasts were significant between W and OES (*p* = 0.007), and OES and SQ (*p* = 0.015)*.

*^b^For TP, contrasts were significant between W and SQ (*p* = 0.0009) and between OES and SQ (*p* = 0.013)*.

**Table 5:** (A) Urinalysis results as a function of time and treatment group in the water (W) group, (B) urinalysis results as a function of time and treatment group in the oral electrolyte solution (OES) group, and (C) urinalysis results as a function of time and treatment group in the subcutaneous fluids (SQ) group.

	AM		Midday		PM	
International units	Mean	SD	Mean	SD	Mean	SD
**A**	**Water**					

Urine specific gravity (USG)	1.029	0.016	1.025	0.015	1.026	0.012
FE Na	0.39	0.42	NA	NA	1.06	0.34
Urine Creat (μmol/L)	13,371	7,541	NA	NA	13,296	8,921
Urine Na (mmol/L)	91.29	97.95	NA	NA	239.17	101.4

**B**	**OES**					

USG	1.034	0.013	1.017	0.01	1.015	0.01
FE Na	0.16	0.18	NA	NA	5.02	3.84
Urine Creat (μmol/L)	15,367	7,284	NA	NA	5,293	6,131
Urine Na (mmol/L)	43.43	52.63	NA	NA	231.33	77.58

**C**	**SQs**					

USG	1.029	0.014	1.027	0.015	1.029	0.014
FE Na	0.24	0.21	NA	NA	1.01	0.32
Urine Creat (μmol/L)	13,721	9,056	NA	NA	14,940	9,408
Urine Na (mmol/L)	42.43	31.25	NA	NA	227.81	103.9

*All values are reported as the mean and SD in international units*.

*FE, fractional excretion; Creat, creatinine*.

**Table 6:** Physical parameters as a function of time and treatment group.

	AM		Midday		PM	
	Mean	SD	Mean	SD	Mean	SD
**Water**						
Body weight (kg)	30.29	4.13	29.86	4.15	30.6	4.27
Pulse (beats/min)	95	10	98	26	100	9
**Oral electrolyte solution**						
Body weight (kg)	30.74	4.35	31.43	4.67	30.56	4.32
Pulse	102	17	96	25	100	15
**Subcutaneous fluids**						
Body weight (kg)	30.56	4.2	30.27	4.1	30.56	4.32
Pulse	100	14	95	22	100	12

*All values are reported as the mean and SD in international units*.

**Figure 2:**
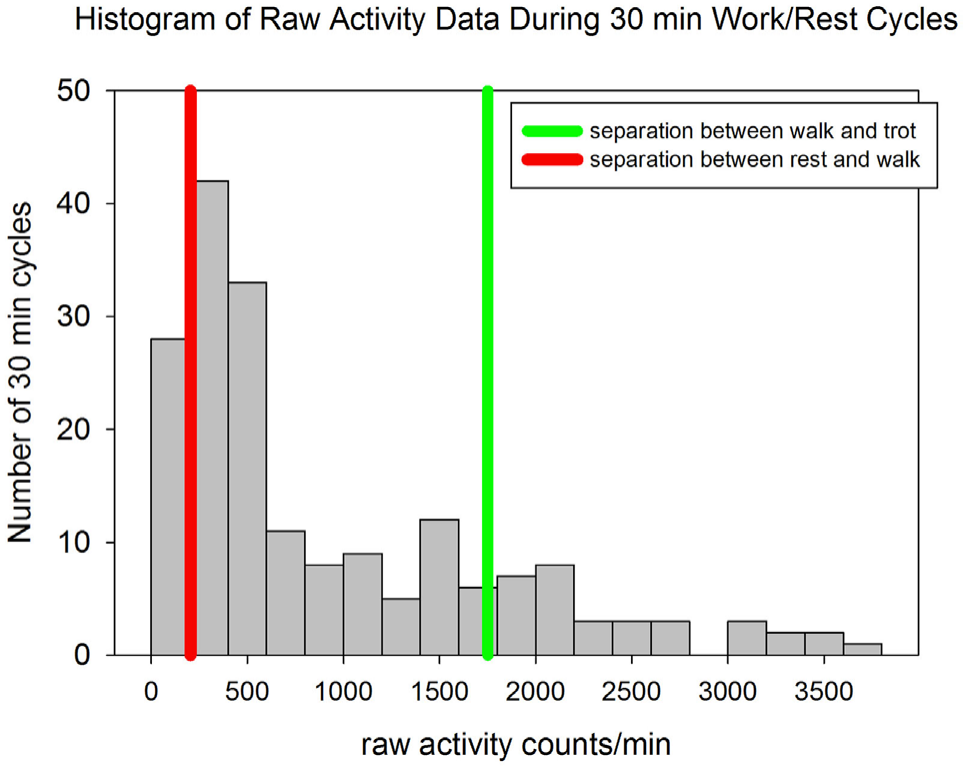
A histogram of the raw activity counts for each 30 min period of either activity or rest for all dogs. The red line represents the activity count of 204 counts/min which has been shown to distinguish sedentary activity from walking activity with 100% specificity and 100% sensitivity in pet dogs. The green line represents the activity count of 1,751 counts/min which has been shown to distinguish walking from trotting activity with 92% specificity and 92% sensitivity in pet dogs (34).

The dogs worked a mean of 5.7 ± 0.8 h per day and their mean activity was 44,975 ± 10,552 counts/kg/h. The dogs spent 15.6% of their time with activity levels consistent with rest (<204 counts/min), 66.7% of their time consistent with walking (activity counts >204 and <1,751) and 17.7% of their time at a trot or more intense level of activity (>1,751 counts/min) (34) (Figure 2). During the study days the median environmental temperature was 84.8°F (29.3°C) (range 74.0–99.9°F; 23.3–37.7°C). The median humidity was 70% (range 39–100%) with a median wind speed of 5.6 mph (range 0–18°mph).

### Effect of Hydration Method

Fluid consumption was influenced by the hydration strategy (*p* < 0.001) but not by the individual dog (*p* = 0.088). Mean fluid consumption in mL/kg/h was 7.04 ± 3.42 for W, 15.61 ± 4.47 for OES, and 5.56 ± 4.40 for SQ. The average additional sodium provided for the OES group was 416 mmol, for SQ was 65 mmol, and for W was 0 mmol. Only dogs in the OES group had the opportunity to consume OES. For those dogs, the ratio of OES to total fluid consumed ranged from 19 to 94%.

When controlling for other variables in the refined models, hydration method had a statistically significant impact on the change in blood TCO_2_ (*p* < 0.0001), Creat concentration (*p* < 0.0001), PCV (*p* = 0.001), and TP (*p* < 0.0001), but had no significant impact on the change in lactate, Na, K, Cl, iCa, BUN, Glu, Hct, TP, and fractional excretion of Na. For TCO_2_, contrasts were significant between W and OES (*p* < 0.0001) and between W and SQ (*p* < 0.0001) with regression coefficients of 0, −2.07, and −1.92 for W, OES, and SQ, respectively. The TCO_2_ in dogs receiving OES or SQ was significantly higher at the end of the day when compared to the W group. For Creat, contrasts were significant between all three pairs of hydration types: W and OES (*p* = 0.015), W and SQ (*p* = 0.010), and OES and SQ (0.001). Regression coefficients for hydration types were 0, −0.13, and 0.12 for W, OES, and SQ, respectively. The Creat in OES although mildly increased from baseline at the end of the day was lower compared to W, whereas in SQ it was higher. For PCV, contrasts were significant between W and OES (*p* = 0.007), and OES and SQ (*p* = 0.015), with regression coefficients of 0, 0.64, and 0.04, respectively. Both OES and SQ treatments resulted in lower PCV at the end of the day compared to W. For TP, contrasts were significant between W and SQ (*p* = 0.0009) and between OES and SQ (*p* = 0.013), with regression coefficients of 0, –0.07, and –0.31 for W, OES, and SQ, respectively. Compared to W, TP decreased more in OES, and SQ groups at the end of the work period.

When evaluating all groups, there was a relationship between fractional excretion of sodium and sodium load (*p* = 0.0001). None of the other parameters tested in the model were significant. When evaluating the effect of time on fractional excretion of sodium for all dogs and all treatment groups, there was a significant increase over the working day (median 0.125 in the AM versus 1.240 in the PM; *p* < 0.001).

### Odor Detection Performance

The variability in duration of search was a function of the difficulty of the search, the environmental conditions, and the type of training aid. The duration of search did not provide data that could be compared across dogs or dates since not all dogs performed the same search. In order to evaluate for fatigue or stress, the behavioral variables were analyzed. Hydration strategy had no effect on any of the behavior scores. Higher activity level was associated with decreased frequency of ears down (*p* < 0.003), stiff tail (*p* < 0.0001), proportion of time the mouth was coded as either fully open or fully closed (*p* < 0.05), panting (*p* < 0.05), and jumping up (*p* < 0.0001). Sex was associated with relaxed ears (*p* < 0.05), stiff tail movement (*p* < 0.005), canine focus on search (*p* < 0.001), lack of reward upon indication (*p* < 0.01), and placing feet up (*p* < 0.01). Females were less relaxed, less focused and less interactive. The breed was associated with duration tail wagging (*p* < 0.05), and canine jumping up (*p* < 0.001) with German shepherds having longer duration of tail wagging and less frequent jumping up than Belgian Malinois. An increased ambient temperature was associated with the canine placing its feet up during reward (*p* < 0.001) and a loose grip on the reward (*p* < 0.01).

## Discussion

In this study of low to moderate activity in detection dogs screening vehicles for narcotics or concealed humans in the Rio Grande Valley Sector (Sarita, TX checkpoint) of the southern US border, the three different hydration strategies had minor effects. There was no detectable effect on internal body temperature or activity. The most dramatic difference between the groups was fluid intake. When dogs were offered the chicken flavored OES, they drank significantly more liquid than when they were in the W or SQ groups. In a previous study of a different OES formulation in search dogs, dogs consumed minimal amounts of OES (35). The OES in this study was readily consumed by the dogs that could have been influenced by the chicken flavoring, the glucose, or the sodium content of the OES.

The rationale for providing OES in humans is based primarily on the electrolyte loss from sweating during exercise. In dogs, sweating is not a major mechanism of heat regulation and sweat glands are primarily localized to their feet (36). This aspect of canine physiology has led some authors to suggest that OES is not appropriate and may not be safe for use in dogs (37).

Dehydration could be expected to increase both PCV and TP through hemoconcentration secondary to water loss; however, both the OES and SQ groups had a significant decrease in PCV and TP. Steiss (38) documented a decrease in PCV over the course of a field trial competition in Labradors. In sled dogs (16), reductions in both PCV and TP have been reported. Expansion of plasma volume during exercise has been proposed to explain the reductions in PCV and TP (16). The TCO_2_ in dogs when receiving either OES and SQ group was significantly higher than when they received W. Both OES and SQ included a buffering agent which may have contributed to the effect on TCO_2_. Alternatively, plasma volume expansion may have resulted in increased perfusion and heat dissipation contributing to less panting (loss of CO_2_) or less of a metabolic acidosis. Blood pH and partial pressure of CO_2_ were not available.

Compared to W, the OES treatment was associated with a smaller post exercise increase in blood Creat; whereas SQ treatment was associated with an increase in Creat. These results suggest that either dehydration or muscle damage over the day was occurring in the SQ group; whereas the OES group had evidence of less dehydration or alternately underwent diuresis. Most Creat originates from muscle stores of phosphocreatine. Small transient increases in Creat may be seen after consumption of a meat based diet, but this should not have been influenced by the hydration strategy as all dogs received the same amount of canned food with the temperature monitoring capsule. In a study of exercising search and rescue dogs provided ad lib water for rehydration, the dogs also had a small but significant increase in Creat (26). Increases in Creat can accompany physical conditioning, exercise, muscle damage (e.g., rhabdomyolysis) or dehydration. In a study of sled dogs with exertional rhabdomyolysis, Creat was not different between affected and controls (39). Rhabdomyolysis is unlikely, although creatine phosphokinase (CPK) was not measured in these dogs. These data suggest that the OES dogs had less muscle injury, were either more hydrated, or were undergoing diuresis.

Although hydration strategy was not a significant factor influencing the difference of fractional Na excretion, the OES group did have the highest Na load. There were no significant changes in blood sodium as a function of time of the day or hydration strategy in the dogs in this study; however, there was a significant increase in fractional sodium excretion in the urine as a function of time of day. Although these dogs were not performing endurance activity, there are not studies of fractional sodium excretion in moderate exercise. The significantly reduced sodium in sled dogs following 10 days of endurance racing (16, 17) raises the possibility of increased sodium loss with prolonged exertion. In one study (16), arginine vasopressin (AVP) was decreased at 2 h after completing the race. In that study, fractional excretion of sodium was decreased post-race, however the authors hypothesized that there was increased urinary sodium loss from activation of the renin–angiotensin–aldosterone system and increased muscle catabolism leading to increased urea filtration (16). In one study of sled dogs, sodium supplementation was associated with improved sodium homeostasis (17). Dogs in all groups had an increase in fractional excretion of sodium over the working day, but maintained their blood sodium concentrations. These findings suggest that despite some sodium loss with work, the normal homeostatic mechanisms in healthy dogs can maintain sodium and handle the additional sodium load from a sodium rich OES. Despite the high consumption, there were no electrolyte abnormalities, suggesting that the OES solution tested was safe in these healthy dogs. Electrolyte supplementation in small quantities may be beneficial in exercising dogs, but further studies are needed.

The influence of dog specific factors (i.e., fluid intake, sex, and breed) as well as environmental factors (i.e., ambient temperature) demonstrates the complexity of hydration regulation. The application of these findings to dogs that perform high intensity work in hot and humid environments may be limited for a number of reasons. The dogs were accustomed to both the environment and steady work load. The mean activity of these dogs was 44,975 counts/h or 750 counts/min; this is 41% of what was observed in working dogs participating in a 30 min exercise protocol (unpublished data). In a study of pet dogs using this accelerometer, rest resulted in a median activity count of 20 counts/min, walking resulted in a median activity count of 1,196 counts/min and trotting resulted in 3,027 counts/min (34). The dogs screening vehicles spent approximately half of the time working but only 16% of their time “at rest,” so throughout the day were active >80% of the time. Extrapolation of these results to dogs from cool climates responding to a disaster in hot climates or being shipped to a military installation in regions with higher heat and humidity may not be accurate. The hydration demands and response to different strategies may also be influenced by the duration and intensity of exertion by the dogs. The variability in the body weight measurements could have been influenced by fluid intake, loss of feces, or urine or variation in the scale. The temperatures and humidity did not reach the extremes that might be present in the most adverse working environments. In addition, the dogs worked under a shaded cover when screening vehicles; therefore, they did not have the effect of direct heat. The mean working temperature in these dogs was 101.9 ± 1.3°F (38.8 ± 0.7°C) which is lower than temperatures typically observed in working, and hunting dogs during exercises which averages over 40.6 C (105.0 F) (6). The inability to document a functional impact of the different hydration strategies on olfaction was limited by the practical nature of a field study in which the dogs were required to perform their normal tasks of screening vehicles. It was not possible to conduct a standardized odor detection assessment with all dogs under the same conditions. The behaviors of the dogs while searching were not affected by the hydration strategy, which may be an indirect measure of performance.

In summary, under these field conditions of controlled activity in moderate heat and humidity, dogs that are accustomed to the work and the environment were more likely to increase fluid consumption when provided a flavored OES. It is unknown if the electrolytes in the OES were beneficial or the effects were simply a function of increased consumption. Under these conditions, indirect measures suggest improved hydration with OES and SQ; however, Creat was increased to a significantly greater extent with SQ. Searching behaviors were independent of hydration strategy but highly related to the dog specific factors of sex, breed and activity level.

Future, laboratory studies under controlled environments may be valuable in addressing the effect of hydration strategy on total body water, the mechanisms of increased fractional sodium excretion with moderate exercise and the impact of hydration strategy on olfactory performance. A laboratory study would also allow for a Latin square design to better control variables. Future field studies are needed to evaluate more extreme conditions, the influence of flavoring versus electrolyte supplementation, and a larger cohort of dogs; but based on the current study, no adverse effects were documented for any of the hydration strategies tested. The use of other OES formulations or the impact of electrolyte-free flavorings was not evaluated and, therefore, the safety and efficacy of these strategies remains unknown. The decision on which strategy is used to best maintain hydration in dogs working in adverse environments will be influenced by the local limitations and demands of the environment and mission.

## Ethics Statement

All protocols utilized were reviewed and approved by the University of Pennsylvania and US Army Medical Research and Materiel Command Institutional Animal Care and Use Committees (U Penn IACUC protocol 804293, USAMRMC proposal SO120002).

## Author Contributions

CO designed the study, executed data collection, reviewed the data, and participated in the manuscript preparation. EH performed the data analysis and participated in manuscript preparation. JN, SP, KK, TD, and KS participated in data collection and manuscript preparation. DC participated in data analysis and manuscript preparation.

## Conflict of Interest Statement

The authors declare that the research was conducted in the absence of any commercial or financial relationships that could be construed as a potential conflict of interest.

## Supplementary Material

Video S1 A video demonstrating a dog performing a systematic search of vehicles and the reward on identification of the odor source. This is a dog in training and with permission of the Penn Vet Working Dog Center. No videos from the study are included in order to preserve the anonymity of the CBP agents.
